# Evolutionary ecology of language origins through confrontational scavenging

**DOI:** 10.1098/rstb.2021.0411

**Published:** 2023-03-13

**Authors:** András Szilágyi, Viktor P. Kovács, Tamás Czárán, Eörs Szathmáry

**Affiliations:** ^1^ Institute of Evolution, Centre for Ecological Research, Konkoly-Thege Miklós út 29-33. 1121, Budapest, Hungary; ^2^ MTA–ELTE Theoretical Biology and Evolutionary Ecology Research Group, Eötvös Loránd University, Pázmány P. s. 1C 1117 Budapest, Hungary; ^3^ Center for Conceptual Foundation of Science, Parmenides Foundation, Hindenburgstrasse 15, 82343 Pöcking, Germany

**Keywords:** evolution, origin of language, confrontational scavenging, evolutionary ecology, agent-based lattice model

## Abstract

A dynamic model and an agent-based simulation model implementing the assumptions of the confrontational scavenging hypothesis on early protolanguage as an adaptive response of *Homo erectus* to gradual change in their habitat has been developed and studied. The core assumptions of the hypothesis and the model scenario are the pre-adaptation of our ancestors to occupy the ecological niche that they constructed for themselves by having evolved displaced communication and a rudimentary tool manufacture, two features allowing them to use a new, concentrated and abundant resource—megafauna carrion—on the savannahs replacing arboreal habitats owing to the drying climate of East Africa at about 2 Ma. The shift in diet required coordinated cooperation by the hominin scavengers confronted with concurrent predators. Power scavenging compelled displaced symbolic communication featuring a limited semantic range; syntax was not yet required. We show that phenotypic evolution on the accuracy of information transfer between cooperating hominins is a necessary and sufficient condition for the population of agents to survive the diet shift. Both the individual and the group fitness of the hominin horde increased with the accuracy of their protolanguage, with decreasing time allocated to foraging and thus more time left for culture.

This article is part of the theme issue ‘Human socio-cultural evolution in light of evolutionary transitions’.

## 1. Introduction

No other species on Earth is equipped with a means of communication even remotely as powerful and flexible as that of *Homo sapiens*, but this is about the only statement that students of different aspects of the origin of language seem to agree upon [1,2]. The many radically different hypotheses on the course and causes of language evolution in human ancestors are rooted in diverse and sometimes mutually exclusive assumptions about the timing, the driving forces and the actual constraints of the process, ranging from the fairly recent and abrupt appearance of an innate ‘language organ’ readily deploying the syntactic rules of a universal grammar in the brain of a late mutant hominine already capable of abstract thinking [2], through 2 Myr of gradual evolution of the mind ultimately featuring symbolic representation and abstraction, language being a byproduct of the evolving mental apparatus [3], or selection for coping with social issues of different kinds within early hominin populations [4], to mention just a few of the many scenarios proposed.

From a biological point of view, it seems obvious that the language faculty, whatever it consists of, resides in the human brain, with a heritable (genetic) basis that is unique to our species [5–7]. The human brain is not only much bigger but also more complicated than those of our relatives: the cortex does look different, and there are human-specific neurons and activity patterns [8,9]. However, these facts do not tell us how the language faculty is structured or whether any specific feature of, say, grammar is innate or not, but at the minimum, they point to original *procedural capacities* in the hominin lineage. Even ‘socialized’ apes have never shown the ability to communicate or reason at our level, which indicates limits to the malleability [10] of the ape brain. Our knowledge of the neocortical and other structures enabling language competence is fragmented and limited [11,12], but neural network modelling, for example, might soon provide novel insights [13]. Even so, the questions why language evolution has taken place at all, and why exactly in the hominin lineage and nowhere else on the tree of life, remain to be answered [14,15]. With regard to the evolutionary takeoff of the language faculty, the question boils down to the contribution of a rudimentary language to the biological fitness of the communicating agents. In short: was there a positive effect of any primitive symbolic communication on the fitness of an early hominin originally lacking it?

The answers are highly context-dependent. Whether symbolic language evolved in the first place as a tool for the mental representation (modelling) of the ‘outside world’, in which case individual selection for the most accurate representation was the primary force driving the evolution of the mind, or as a means of sufficiently accurate information transmission among individuals of a hominin group to aid effective (synergistic) cooperation [16], is difficult to determine. But the potential fitness effects on the individual and the group level are not mutually exclusive. The actual driving force can be seen as the combined effect of individual and group advantages, possibly with shifts in their relative importance during the course of hominin evolution, suggesting a possible coevolutionary scenario for the simultaneous origin and development of human cooperation, language and mind.

The present study aims at testing the feasibility of potential selective advantages for a mating group from evolving a simple protolanguage to coordinate within-group cooperation in power scavenging in an early hominin population. The conceptual starting point for our agent-based model is the confrontational scavenging scenario [17,18] which is based on ecological theory and palaeo-anthropological, palaeontological and behavioural evidence. The main argument of the hypothesis is ecological: habitat shift (from forest to savannah) owing to climate change about 2 Ma forced early *Homo erectus* groups to radically change the main resource they subsisted on. Their survival depended on occupying a niche they constructed for themselves by becoming able to process large herbivore carcasses before they could be consumed by competing animal scavengers. Possessing Oldowan lithic technology (sharp stone flakes suitable to cut through very tough hides—one of the key factors in the construction of the new niche), *H. erectus* may have had access to a large amount of meat in a mega-herbivore carrion days before it exploded owing to internal gas production and became available to concurrent scavengers lurking around. There is isotopic evidence that the proportion of meat consumption increased following the origin of *H. erectus*, most markedly around 1.65 Ma [19–21], a view strengthened by palaeoecological, archaeological and anthropological analyses [22–24]. The supply of mega-herbivore carcasses may have been indeed large compared with the density of early hominin scavengers subsisting on them: the number of 2-million-year-old hominin mandibles found in the Omo–Turkana Basin is more than two orders of magnitude smaller than the pooled number of mandibles from the same time of Giraffidae, Elephantidae, Hippopothamidae and Bovidae, many species of which are large herbivores with hides initially impenetrable for scavengers or predators other than *H. erectus* [25], and the rest still large enough to be worth the cooperative effort necessary to confront other scavengers to possess them. Making use of this advantage, i.e. occupying the new niche thus created, required extensive and coordinated cooperation from early hominin populations. Cooperation and coordination were necessary in at least two contexts in order to exploit the rich resource the expanding savannah had to offer for a horde. First, *displaced communication* was necessary for the recruitment of sufficiently large teams of butchers by letting them know of the existence, the size and the distance of the find outside the sensory range of the horde. Second, to process and transport the carcass without a very high risk of being robbed of it by, or falling victim of, concurrent predators required coordinated defence of the group. The confrontational scavenging hypothesis posits that the evolutionary pressure to such an effective coordination of cooperative actions through abstract signalling may have been the initial selective force for the development of protolanguage.

In order to provide an eco-evolutionary basis for the confrontational scavenging scenario, we proceed in two steps. First, we present an individual-based and spatially explicit model that illustrates in considerable behavioural and energetic detail how the scenario unfolds as a consequence of elementary communication, recruitment, scavenging, cooperation and food sharing, without hard-wiring in many a detail so that the outcome can be justifiably regarded emergent. Second, we introduce a deliberately simple semi-analytic model taking into account alternative strategies, group formation and costs and benefits of cooperation (see electronic supplementary material, §S2). This model highlights some key features of the evolutionary dynamics. Our main question is whether the resource shift induced by climate change enforces an evolutionary improvement in the accuracy of displaced communication in the hominin group. The question can be reformulated from a more general evolutionary ecological perspective: How efficient could our early ancestors have been in constructing and occupying their own ecological niche in the confrontational scavenging scene, through evolving protolanguage as a means of coordinating cooperation? We also ask how the resource shift affects the efficiency of foraging and the survival of the horde with and without evolving communication.

## 2. Materials and methods

### (a) Model basics

The model follows the fate of a single simulated horde on the individual level over several thousand generations on an hourly basis. There are two types of hominin agents in the model: ‘adults’ and ‘children’. For the sake of simplicity, genders are not specified in either group. At any point in time the current number of adult individuals is denoted by *N*; the maximum number of the adults of the horde is *N*_max_ = 100; the maximum number of individuals (including children) is *N*_all_ = 1000. This overall group size probably exceeds that of a *H. erectus* horde at the time. We have set *N*_all_ high for technical reasons: too small group sizes make the dynamics erratic, requiring long series of simulations with many replicate runs; larger populations allow a single representative run with each parameter set. (All parameters of the model can be found in the electronic supplementary material, table S1.) The habitat is represented by a 120 × 120 rectangular grid; the size of each cell is 0.25 × 0.25 km, so that the spatial extension of the grid is *L* × *L* = 30 × 30 km^2^. The central cell of the habitat is the campsite of the horde, a safe place for the members of the horde to sleep, communicate, share food and form cooperating groups. The distance of each location is measured relative to the campsite. The habitat offers two types of resource for the horde: ‘berries’ and ‘carrion’, the latter meaning the carcasses of dead mega-herbivores. ‘Berries’ may be anything readily available for consumption by a solitary hominin agent at the spot of discovery, including plants and small animals not requiring cooperation to harvest. Individuals may search for food by moving on the grid during the 12 h daylight period; they must return to the campsite by sunset at the latest. In the morning, every individual engages in one of the following three activities: (i) stays at the campsite; (ii) leaves the campsite in search for food (berries or carrion) alone or (iii) joins a butchering team leaving the campsite to process and bring in a mega-herbivore carcass previously found by a solitary forager. Solitary foraging is performed by a correlated random walk in which the probability of a forward step is 0.92; the probabilities of turning left or right are 0.04 each. The speed of walking depends on the amount of the resource carried. The maximum speed is *v*_max_ = 2.5 km h^−1^ (with which an individual can cover 30 km a day: the distance to the end of the habitat range and return). Our assumption of an action radius of around 15 km follows data and reasoning based on archaeological and palaeoecological evidence and inferences [23]. Worthy of note is that the parallel acquisition of meat and stones shows that visiting a number of regular sites is energetically preferable to foraging on-the-go, as shown by an early simulation antecedent [26]. More specifically, we favour the evidenced assumption of focal sites, in adherence to the resource-defence model, with multiple features (sleeping, water, trees, plants and other, smaller food items) [27]. The food load decreases the speed linearly:

$$v(E_{\mathrm{carry}})=v_{\max}\left(1-\frac{E_{\mathrm{carry}}}{2000}\right),$$

where *E*_carry_ is the energy content of the carried food in energy units, the universal ‘currency’ of all types of resource (see §2b). Note that the maximum daily walking distance of 30 km (15 km outward and 15 km inward) can be covered only by individuals not carrying anything. The maximum amount of energy potentially carried by an individual in the form of meat is *E*_meatmax_ = 1000 units; with berries it is *E*_berrymax_ = 800 units, the difference being due to the lower energy density of berries (see §2b). Carrying the maximum load reduces walking speed to $v_{\max}/2$, so that the maximum distance possibly covered by loaded walking in *t* hours is a linearly decreasing function of the actual load from *v*_max_ *t* to (*v*_max_*t*)/2. This has to be taken into account when planning meat transport: butchering teams have to return to the campsite before night falls with as much meat as possible. For efficient planning, the size and the distance of the carcass have to be estimated by the scout who finds it. A distinction between butchery and consumption sites prevailing at about 1.75 Ma was identified at the Olduvai Gorge, showing signs of early access to carcasses before other scavengers, efficient butchery including defleshing and secondary butchering of selected carcass parts as well as consumption of meat and marrow at the camp site [28]. Also, another study of the time sequence of cut marks on bovine bones from the Olduvai site suggests that scavenging was probably the dominant way of obtaining meat at the time [23]. We assume that these data can be accurately assessed by any adult member of the horde in rough categories using three different carcass size classes (small, medium and large) and two distance classes (near and far); see details in §2b. The maximum amount of energy (*E*) that can be carried back by sunset is a function of the actual distance from the campsite (*s*) and the remaining daylight hours (*t*) as specified by the following equation:

$$E(s,t)=\left\{2000\left(1-\frac{s}{v_{\max}t}\right)\right\}\;,$$

where *s* is measured in kilometres, *t* in hours and {...} represents clipping into the [0, *E*_berrymax_] range in the case of berries and the [0, *E*_meatmax_] range in the case of carrion. Note that the default value of the maximum amount of meat carried per head (*E*_meatmax_ = 1000) is half of the maximum energy reserve of an adult agent (*E*_max_ = 2000—see §2b); so the maximum meat load represents about a quarter of the body mass of an agent (assuming the maximum energy reserve is contained in about half of their body mass).

### (b) Energetics and environment

For the sake of simplicity, harvested and consumed resources, as well as the actual energy reserves of each individual, are measured in arbitrary units of energy. Also, the actual values of the model parameters of energy intake and utilization are arbitrary, but consistent with the results of studies estimating the corresponding data for *H. erectus* (e.g. [29–32]). The current energy level of an individual is denoted by *E*, the maximum of which is *E*_max_ = 2000 units. Individual energy balances are increased by eating food and decreased by a few different factors. A single meal can increase the balance at most by *E*_eatmax_ = 200 units in adult agents and by 100 units in children, the actual increase depending on the amount consumed. A solitary individual can harvest and eat berries on the spot of the find instantly. The members of a butchering team can eat from carrion wherever it is found; they can also eat from the food (berries or carrion) they bring into the campsite in the evening, and from the remaining food (if any) the next morning—see details later. The daily energy use of an idle agent is *E*_daily_ = 100 units day^−1^ for adults and half of that for children. There is an energy cost for movement: *E*_move_ = 1 unit per 12 min of walk (notice that this takes the effect of load into account: higher load lowers walking speed and thus increases transporting time). Communication has a small daily cost as well: *E*_comm_ = *κC* units day^−1^, where *C* is communication ability (0 ≤ *C* ≤ 1) and *κ* = 10 is a factor of proportionality.

Each grid cell, except for the campsite, may contain one of the two types of food source (a berry bush or a mega-herbivore carcass). The total number of carcasses within the habitat is *F*_C_, and that of bushes is *F*_B_ = 1500, the difference representing the higher energy density of carrion. The default value is *F*_C_ = 100; this is one of the main parameters of the model. Berries on a bush are replenished at a rate of *R* = 300 units day^−1^. The maximum energy content of a bush is 300 units, while the energy content of carcasses follows a uniform distribution drawn from the range [60 000, 90 000] units, representing 30 to 45 times the energy reserve of an adult hominin, i.e. the energy content of a carcass is 15 to 22.5 times larger than that of a hominin agent. This amounts to a mass of meat of about 1 metric tonne per carcass, which is consistent with the size range of mega-mammalian species [33]. This range of carcass sizes is arbitrary, but not far from more realistic estimates (used, for example, in the HOMINIDS model: [34]. Berry bushes might change location (one disappears, another one appears) every 130 years on average.

Scouting and foraging individuals move with the same directed random walk pattern. Every forager is a potential scout, returning with the information (size and distance) of a carcass when one is found. Foragers walk around eating berries until they are full, then gathering as much as they can carry or until they have to return home for the night. This means that in the carrion phase the scouts are still foraging if they happen to stumble upon a berry bush still existing (10% of bushes, each with 10% yield remain, with a total berry supply of 1% of the starting berry-only phase all along the carrion phase). A butchered carcass is available for others (a scout or another group) for 3 h. After that time the carcass is occupied and eaten by carnivores. (This usually happens in the rare case of more than one butchering team arriving at the same carcass on the same day or an unlucky scout stumbling upon a freshly butchered carcass on the way home at dusk.)

Butchering teams may take as much meat home as their members can carry at most; if the carcass processed is larger than the transport capacity of the team, the remainder is left on the spot and either consumed by concurrent scavengers during the night or may be carried back by another team arriving during the same day, within 20 min after the first (butchering) team has left. A butchering team finding a carcass already fully exploited moves around in a random pattern until either they find a non-butchered carcass or they run out of action time and head home for the evening. Intact carcasses remain available for the hominin scavengers for 3–6 days (cf. the data in [33], for carcass persistence times of recent mega-mammals), after which they become accessible for concurrent scavengers and disappear. A carcass, whether it is consumed or not, is replaced by another one at a random site after 3–6 days.

Energy uptake (eating) follows simple rules. A solitary gatherer finding a bush eats berries to satiation (*E*_eatmax_ = 200 units) and carries home as much of the leftover as they can. If the current bush is exploited, the individual continues looking for new bushes. A gatherer becomes a scout if they stumble upon an un-butchered carcass that will not disappear by the next day. The members of a butchering team eat *E*_eatmax_ = 200 units each on the spot and carry back home as much as is left and does not exceed their collective transportation capacity. (Note that the load reduces their speed of walking and increases their energy consumption since it takes longer to get back.) Upon arrival at the camp every butchering team shares the food brought home, first among themselves up to satiation (200 units per person), with the team leader's quota 50% higher than that of the others. (The extra 50% provides an advantage only in the case of low supply.) The surplus, if any, is then distributed among the rest of the horde: children are fed first, from oldest to youngest (max. 100 units); if there remains still more food, it is shared equally among adults who are still hungry.

The effect of climate (and habitat) change is implemented as a gradual shift from one food source to another: from berry to carrion. At the initial phase of the scenario, there are only berries available; in the mixed phase, both the replenishment rate and the number of bushes decrease linearly to 10% of their initial values during *τ* = 10 000 years—the transition phase—resulting in 1% of the original total supply. Parallel with the decrease of the berry supply the amount of carrion meat increases linearly from 0 to *F*_C_ carcasses. In our model, the transition phase starts at year 20.000 and ends at 30.000. The size distribution of the carcasses does not change during the transition phase and the carrion phase.

### (c) Communication and group formation

Successful scouts (those who have found carrion) attempt to recruit butchering teams to process and transport the meat home during the next day. The optimum size of the group depends on both the size of the carcass (small, medium or large) and its distance from the campsite (near or far). The optimum team size is assessed by the scout based on the information that they obtain on the spot; recruitment continues until the supposed optimum is reached or there are no candidates left. The actual values used in the simulations can be found in electronic supplementary material, table S2.

The information on which the decision of the recruits on joining a team is based is the risk of death associated with the actual mission, the exact measure *D* of which is proportional to the distance of the carcass from the camp: *D* = *ds*, where *s* (km) is the distance and $d=1/15\;{\mathrm{km}}^{-1}$, a factor of proportionality. The effect of risk dilution (the effect of risk-sharing within the team) is neglected in risk assessment for simplicity—again a conservative assumption. The maximum level of actual danger (*D* = 1) corresponds to the maximum distance (approx. 15 km), from where only a very small chunk of carrion can be brought back to the camp. The information on the actual risk of joining a butchering team $\left(\tilde{D}\right)$ perceived by the observer of a recruitment performance is distorted by the joint inaccuracy of speaking by the scout and understanding by the potential recruit. The level of danger $\left(\tilde{D}\right)$ perceived by a listening recruit is dependent on the product of his own personal communication ability and that of the speaking scout: *C*_speaker_*C*_listener_. We assume that the same individual is just as good a speaker as he is a listener. Thus, the total distortion on the communicated danger is

$$\rho =(1-C_{\mathrm{speaker}}C_{\mathrm{listener}})U(-1,1),$$

where *U*( − 1, 1) is a uniformly distributed random number from the [ − 1, 1] interval. This choice guarantees that *ρ* = 0 only for *C*_speaker_ = *C*_listener_ = 1, and it can be large if *C*_speaker_ = 0 and/or *C*_listener_ = 0. The perceived distorted level of danger is $\tilde{D}=\{D+\rho \}$, where {...} denotes clipping into the [0,1] range.

A listener can join the butcher team being recruited if his perceived risk $\tilde{D}$ is lower than his current ‘bravery’ (*B*). Bravery is determined by hunger: $B=1-(E/E_{\max}),(0\leq B\leq 1)$, i.e. hungry individuals are willing to take more risk. Hungry children linearly increase the bravery of the adults up to 10% if every child is left hungry at the end of the day. A motivated individual with communication ability *C_i_* joins the team with probability *C_i_*. This makes group formation stochastic (in contrast with a possible deterministic algorithm simply sorting the applicants by their actual motivation to join) and can result in groups of size less than optimal for the mission.

If more than one scout is successful in a single day (i.e. if more than one carcass was discovered the day before), multiple butchering teams are assembled one by one, starting with the best-communicating scout and followed by the others in order of communication skill. After the first team is complete, the next scout starts recruiting, etc. until there remain no more applicants or scouts. A butcher team assembled in the morning sticks together until it returns to the campsite in the evening. The rest of the horde (those not in butchering teams) remain at the campsite or leave for gathering/scouting, depending on their actual motivation level (*B*). An unsuccessful scout (one unable to recruit even a single team member) gives up and becomes either available for recruitment or a scout.

### (d) Population dynamics, ecology and evolution

Children at age 10 reach adulthood and either they join the active core of the horde if there is a place for them (*N* < *N*_max_) or they leave the horde (dispersal). Adult individuals die with a constant $\delta_{\mathrm{nat}}=10^{-4}\;{\mathrm{day}}^{-1}$ basic death rate (which corresponds to an average lifespan of $\tilde{l}=1/(365\;\delta_{\mathrm{nat}})\approx 27.4$ years, excluding other mortality factors). Individuals may also die owing to starvation (if their internal energy reserve level *E* hits 0, which occurs to an initially well-fed agent failing to obtain food for 20 consecutive days) or to carnivore attacks while butchering and transporting meat to the campsite. The death rate from carnivore attacks is proportional to the distance *s* between the carcass and the campsite: $\delta_{\mathrm{move}}=(8/3)\times 10^{-5}\;(km^{-1})\;s\;(\mathrm{km})$. People stop breeding and scavenging at an age of 40 if they live that long. Childbirth is possible only if the energy reserve dedicated to offspring production by the parent reaches at least 32 000 units. Newborn children start their extrauterine lives with an energy reserve of 600 units. Each fertile parent can have a viable child as soon as they have accumulated sufficient energy for a pregnancy, which takes 14 months at the maximum of food intake rate and more at lower resource intake. A child inherits the communication trait *C* of the parent with an additive phenotypic mutation term drawn from a normal distribution with 0 mean and *σ* variance.

The daily routine of the horde is the departure of butcher teams and gatherers/scouts in the morning, who all return by sunset. The evenings are spent sharing the food retrieved during the day, telling stories about new food sources discovered, and the recruitment of tomorrow's butchering teams. An animated visualization of what actually happens in the habitat of the horde is shown in electronic supplementary material, movie S1.

## 3. Results

Since the scenario is relatively detailed, the model has a large number of parameters (see electronic supplementary material, table S1 listing all the parameters and their default values), but most of them are fixed by the spatio-temporal scale of the process or constrained into narrow ranges of values by straightforward assumptions. The rest of the fixed parameters regard the energy balance of the hominin population and the environment. These are basically set by an arbitrary scaling assumption, but they are canonical among each other: the arbitrary choice of value for one determines the feasible range of the others. The only evolving variable of the model is *C*, the accuracy of displaced communication.

**Figure 1.**
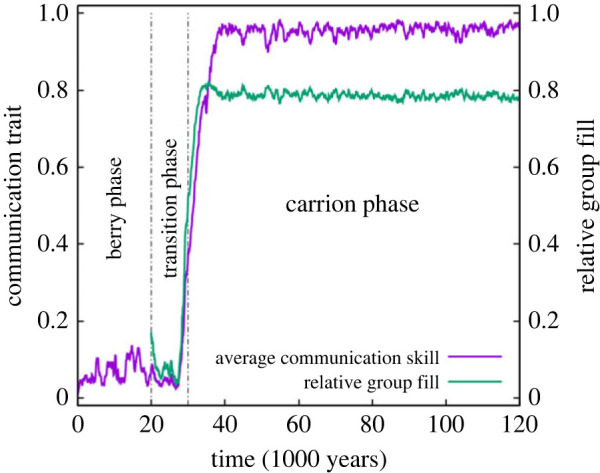
The evolution of the communication trait and team saturation. The population average of the communication trait, i.e. the mean accuracy of communication within the horde is shown in purple. The team saturation is measured in proportion of the optimal team size (green). Vertical dot–dashed lines indicate the start and the end of the climate change period. Parameters are taken from the standard parameter set (*N*_max_ = 100, *F*_C_ = 100, *σ* = 0.01). (Online version in colour.)

Climate change and the consequential resource shift starts at *t* = 20 000 and ends at *t* = 30 000 years in all simulations. These dates mark the borders between the three phases (berries, transition and carrion) of the simulated time period, which covers 120 000 years in most cases (200 000 if needed for reaching a stationary state). The shift in the availability of the two basic resources, i.e. the decrease in berry and the simultaneous increase in carrion supply, initiates an evolutionary change in the communication ability of the hominin agents, but the actual increase of communication skills starts later than the environmental change, and it also extends into the carrion phase. Communication approaches its maximum accuracy in a nearly uniform fashion across the horde (figure 1).

The average energy obtained by a solitary (adult) agent in the berry phase is about 325 units per day, while the *per capita* energy yield of a cooperating scavenger in the carrion phase is a bit higher, approximately 345 units in a day; see electronic supplementary material, figure S1*a*. The difference is small, and it comes from the extra energy that an individual can obtain carrying home a resource of energy density 25% higher, discounting the costs of increased mental capacity and the extra distance covered by the scouts and butchers compared with gatherers in the berry phase. Any extra amount of obtained energy is channelled into generating and sustaining more offspring, which results in a higher young adult outflow (emigration) from the horde during the carrion phase. Electronic supplementary material, figure S1*b* shows that the average energy level corresponding to the average fitness of the individuals increases from 1170 units in the berry phase to about 1250 in the carrion phase. The approximately 10% increase is due to the energetically more favourable situation in the carrion phase (targeted exploitation of high-density food patches).

**Figure 2.**
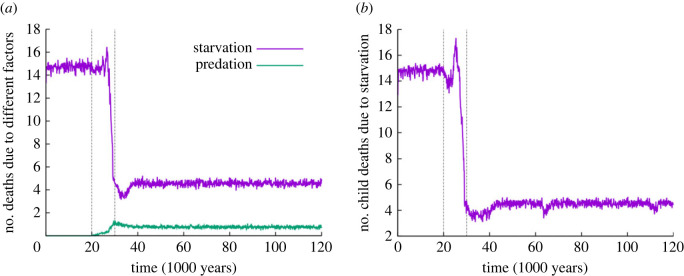
Number of deaths per year by external factors as functions of time. (*a*) Total mortality due to starvation and predation. (*b*) Child mortality due to starvation. Purple: number of deaths by starvation; green: number of deaths by predation. Vertical dashed lines indicate the start and the end of the transition phase (standard parameter set as in figure 1). (Online version in colour.)

Figure 2*a* shows the temporal change in the number of deaths per year due the two external mortality factors (starvation and predation). Deaths per year by starvation are more than three times more frequent in the berry phase (approx. 15) than in the carrion phase (approx. 4.5). Children's death rate due to starvation falls even lower in the carrion phase (figure 2*b*). This difference, along with increased resource availability and the consequential fertility gain in the carrion phase, is the primary selective force driving the transition. Even though the number of deaths due to the second external mortality factor—predation—is small, approx. 1.0), it represents a potent selective force towards the evolution of accurate communication. Misunderstanding the actual level of predation risk during butchering missions is detrimental in both directions: underestimating the risk results in extra predation mortality, whereas overestimating it induces unjustified cowardice preventing optimal team recruitment, and thus causes relative starvation. That is, the death rate due to predation is about half of the cost of selection for better communication skills within the horde (selection load). The other half of the selection load is the indirect loss of resource benefits due to cowardice, which is obviously effective but difficult to quantify. (As the natural mortality rate *δ*_nat_ is constant, the number of deaths is constant during the whole simulation and it is not shown on figure 2.)

The average lifespans of actively foraging adults (individuals older than 10 years) and children (under 10 years) are 22 and 5 years, respectively. By taking only the natural mortality of rate *δ*_nat_ into account the expected lifespan of an adult agent is $1/(365\;\delta_{\mathrm{nat}})=27.4$ years. The decrease in expected adult lifespan to 22 years is due to starvation and predation (external factors), in different proportions in the berry and the carrion phase.

Since mega-herbivore carcasses are relatively large compared with the transport capacity of the average butchering team (see electronic supplementary material, table S2), butchering teams rarely have sufficient personnel to carry all the meat available from a carcass back to the campsite. The average of team saturation (the actual team size relative to the optimum for the given carcass) increases during the transition phase roughly in proportion to the average accuracy of communication within the horde, its average reaching about 80% at the stationary state (figure 1, green curve). Note the parallel change in communication skill and group saturation.

**Figure 3.**
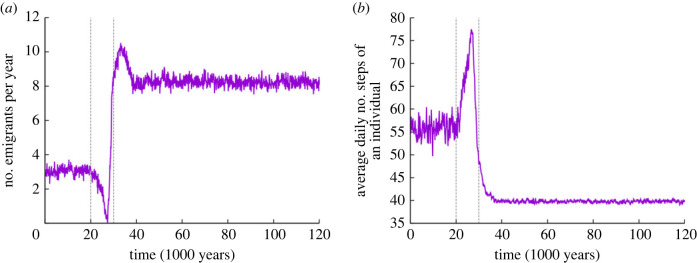
(*a*) Yearly number of young adults leaving the population. If this number drops to zero, then the horde is at or below the replacement rate and will slowly die out. This is different from the classic case of extinction due to starvation and takes several generations. (*b*) Total average daily number of steps taken by an individual. Vertical dashed lines indicate the start and the end of the transition phase (standard parameter set as in figure 1). (Online version in colour.)

The turnover of the population can be measured by the excess (over the 100-adult ceiling of the size of the population) number of young adults (10-year-old individuals) who are forced to leave the horde. Figure 3*a* shows that the output of emigrants by the population is more than three times higher in the carcass phase, a clear indication that switching to the new, energy-rich and patchily distributed resource is an evolutionary success achieved through selection for accurate communication. Note that the peak in the number of emigrants is the result of offspring overproduction at the beginning of the carcass phase—subsequent increase in communication accuracy improves the assessment of optimum risk-taking against the benefit of producing more offspring, many of whom leave the group anyway as emigrants. Producing more emigrants is a typical group-level advantage, but this model is intended to show that communication provides an obvious evolutionary advantage even without group selection taken into account.

An interesting aspect of the transition from berries to carrion as the main resource of the population is the change in the time that an average agent spends foraging. Considering ‘laggards’ with zero foraging time and also the smaller number of steps taken by butchers compared with gatherers, the difference is substantial between the two phases (figure 3*b*). In the carrion phase less time is spent foraging. The remaining ‘free time’ could be allocated to developing new technologies, raising children, socializing and creating culture—all these activities indirectly contributing to both individual and group fitness. This effect is not yet considered so that the free time thus obtained is wasted in the model, but it is safe to assume that the obvious benefits of the time used to create culture could also significantly contribute to human evolution, including the perfection of language skills.

Electronic supplementary material, movies S1, S2 and S3 visualize the activities of the horde in the two phases. Electronic supplementary material, movie S1 animates 15 days in the berry phase, zoomed on the neighbourhood of the campsite. Electronic supplementary material, movie S2 follows 50 days in the carrion phase; electronic supplementary material, movie S3 is a zoomed slow-motion version of electronic supplementary material, movie S2, with relevant events marked.

The standard parameter set was assembled with feasible spatial and temporal scales of the Palaeolithic scene in mind so that the foraging range and the demographic features like the average lifespan and the birth rate in the hominin horde take reasonable values. The parameters characterizing the environment (resource input functions, resource distribution, the rate of climate change) and the time scale of the evolutionary acquisition of protolanguage skills (the phenotypic mutation rate of the accuracy of communication) are relevant and potentially important with respect to the focal question: at what values of these parameters can we expect sustained persistence of the population with displaced communication evolving and spreading under changing ecological conditions?

Electronic supplementary material, §S1.3 gives a detailed description of the actual screening results, which we summarize here briefly. The length of the transition period (*τ*) is indifferent as long as it exceeds a reasonable minimum allowing communication skills to evolve in the horde featuring a certain rate of phenotypic mutations (*σ*) (see electronic supplementary material, figure S2*a*,*d*). At *σ* = 0.005, the minimum of *τ* is about 20.000 years. The total supply of carrion biomass available within the spatial range of the horde (*F*_C_*S*_C_) has to exceed a minimum in order to keep the population viable during the transition and the carrion phases (see electronic supplementary material, figure S2*b*). Average carcass size *S*_C_ (i.e. the spatial distribution of the resource) has a threefold effect on population growth through the chance of finding, butchering and transporting carcasses, as well as on protolanguage evolution through the need to organize all these activities (see electronic supplementary material, figure S2*c*). Electronic supplementary material, figure S3 shows that the main demographic effect of a successful diet shift is the spectacular increase in the steady-state number of children in the horde in the carrion phase. With the size of the horde at its limit, the surplus of juveniles all become emigrants, the number of whom is obviously related to the group fitness of the horde.

We present a family of analytic ‘toy’ models of the scenario in the electronic supplementary material, in which Scavengers (S) and Parasites (P, doing no work) are always present, whereas Gatherers (G) are optional. Results are presented for groups of two and larger (especially three), with or without kin selection and/or special assortment of S by recruitment (assumed to depend on communication), with linear, convex and concave benefit functions. Relatedness and recruitment are always helpful, by either making invasion of the G–P community by S possible, or at least enlarging the basin of attraction for S (with a mutant tail of P). Note that, unlike the simulations above, these models account for infinitely many groups of limited size. Naturally, diminishing fruits and more frequent carcasses help the transition to S.

## 4. Discussion

The question that this model attempted to answer is a simple yes-or-no one: could the acquisition of a rudimentary form of displaced symbolic communication support the persistence of early hominin populations amidst the drastic climate change driving them from their previous arboreal habitat out to the savannah? Our answer to the question is a definite ‘yes’, provided that the assumptions behind the model scenario are correct. Did a large herbivore megafauna indeed spread over African savannahs [35] while forest habitats retreated [36]? Was the time scale of the process of climate change long enough for hominin adaptation to trace it in both the physiological and the behavioural sense? *Homo erectus,* using both Oldowan and Acheulean technology, had the means and the tools [37] at their disposal at the right time to construct the power-scavenging niche for themselves. Researchers have argued meticulously [17,38] that these conditions had been met by about 2 Ma, setting the scene for early humans to occupy their self-constructed niche, mainly driven by group selection for extensive cooperation coordinated by displaced communication.

According to the confrontational scavenging hypothesis, protolinguistic ‘Adam’ belonged to *H. erectus* [17]. It is remarkable that the latest find in South Africa suggests that *H. erectus* is at least about 2.04–1.95 million years old, and that the analysis of other hominin species there and the high taxonomic diversity of also non-hominin species indicate a period of climatic variability [39]. In this setting *H. erectus* faced challenges that exerted strong selection pressure on the population. A recent review explains: ‘Among primates, terrestrial species live in larger groups with more and bigger males than arboreal taxa, particularly those that live in savannah versus forest environments; they also have higher frequencies of counter-attacks against known predators, and if this pattern was followed as early hominins became committed bipeds, it could have led to confrontational scavenging. If hominins discovered carcasses in different locations on the landscape than where the stone needed to process those carcasses were located, as well as in different locations than preferred plant foods, scavenging may have selected for characteristics including social cooperation, planning depth and detailed mental mapping’ [40, p. 11].

Confrontational scavenging must have been intermingled with primary scavenging, hunting of smaller prey, and gathering [40]. A long-lasting trend of disappearing fruits and the dry periods of climatic variability [41] were factors likely to have selected for the first habit. Even endurance running, speeding up carrion acquisition, could have appeared with the scavenging habit, to limit the duration of bacterial growth in the carcass and transported, butchered meat [42]. Significant is the suggestion that cooking on fire might have originated with early *H.* *erectus *at about 1.9 Ma [43], which not only could have allowed for evolutionary brain growth but also, we suggest, further decreased adverse bacterial effects of carcass consumption [44]. A problem with this idea is the late evidence for fire itself, which, however, raises the following questions. First, How could *H. erectus* use increased energy, reduce its chewing efficiency, and sleep safely on the ground without fire? Second, How could a cooked diet have been introduced to a raw-foodist, mid-Pleistocene *Homo* without having major effects on its evolutionary biology? Satisfactory answers to these questions will do much to resolve the tension between archaeological and biological evidence [45]. Confrontational scavenging and the emerging language could have selected for preferred interactions (friendship: [46]).

It is striking how tightly the survival of the population in our model is dependent on the agents evolving communication skills to perfection under any reasonable ecological condition (figure 1). The only parameter combinations allowing less than nearly 100% accuracy of communication at the carrion phase while still ensuring survival are related to the ease of access to carrion supplies (electronic supplementary material, figure S2*c*). Abundant and easily accessible meat supplies make it possible for quite inaccurately communicating agents to survive, either as laggards avoiding participation in butchering missions altogether and subsisting on the leftover which is still abundant, or as members of small teams of butchers running a low risk in butchering and transporting meat because many small carcasses are easy to find close to the campsite. Accurate communication is not indispensable for the ecological success of the horde in times of easy access to resources. On the other hand, the accuracy of communication (*C*) evolves to its highest possible equilibrium value on the verge of extinction, when reliable information about concentrated resource patches (carcasses) and the cooperative actions of large teams are vital to avoid starvation. This explains why the most severe resource shortage that the population can still survive results in the highest accuracy of communication, especially when the scarce food supply is distributed in a few large lumps. The reason for surviving these unfavourable conditions and avoiding famine is precisely the communication skill evolved. The cross-check for the role of displaced communication in population survival is that not allowing *C* to mutate above 10% of its maximum value during the simulations inevitably leads to extinction during the transition phase.

All the variables related to individual and group fitness are improved by the time the transition from the berry to the carrion phase is completed. Of course, this model does not consider any effect of the resource shift on the basic fitness of the hominins due to direct changes in diet or indirect ones through extra time becoming available for manufacturing tools and for cultural activities—factors with likely significant positive effects on life expectancy. What improves with the transition in the model is the average energy level of the agents (electronic supplementary material, figure S1*a*), which results in lower death rates due to starvation, but this improvement is partly compensated by the increase in deaths due to predation during scavenging missions. The latter is the selection load of evolving accurate communication (figure 2). Improved group fitness variables include the number of children supported and the release of superfluous young adults (emigrants) from the horde (figure 3*a*; electronic supplementary material, figure S3)—a possible large-scale side effect of which could have been the first out-of-Africa dispersal wave of *H. erectus* [47].

A different agent-based model [48] found that responding to symbolic but not syntactic vocal signals enhances resource acquisition in a foraging population of agents with phase-transition-like dynamics of resource efficiency similar to what we have observed. A remarkable feature of the considered scenario is the tight link between emerging cooperation and protolanguage. In general, the argument has been made that components of the human-specific adaptive suite (language, cooperation, teaching, tool making and use, complex theory of mind, shared attention/intentionality) could have evolved more readily in combination than one by one [6,7,15] owing to the synergistic fitness advantages provided by the interacting components.

Finally, we make a few remarks on the supposed structure of a minimal protolanguage [1,49] that could be sufficient to serve the transition from a solitary gatherer lifestyle to group cooperation in confrontational scavenging. There are two semantic aspects of communication constituting the minimum condition of successful group cooperation in this context: it must be capable of (i) conveying displaced information about events outside of the sensory range of the listener (telling ‘stories’ about having found valuable resources far away), and (ii) coordinating actions for the defence of the resource and fellow agents during meat processing and transport. Neither of these requires syntax nearly as sophisticated as that of any known language today, even if displaced communication is special in the animal world (bees being another remarkable example). Once that abstraction was in place, even a primitive sign language could do to make it functional under the changing environment of *H. erectus* and ensure the survival of its populations, with vocalizations slowly taking over [50]. The final goal of research on the origin of language is a theory explaining how and why syntactic languages have evolved in our species, but on the basis of our model's predictions, we support the proposition [17] that a syntax-free protolanguage may have been the point of departure for that long evolutionary process. An exciting experiment [51] with communicating human agents confirms that syntax is not essential for the coordination of simple cooperative tasks to be surprisingly efficient—all that is needed for harvesting most of the benefits of cooperation is abstraction and a very limited vocabulary. Following phases of language evolution—including the invention of syntactical rules to accurately transmit subtle message details—may have been built on the solid, ecologically founded evolutionary advantage of using a protolanguage. The uniqueness of natural language is explained by the fact that protolanguage evolved into syntactical language in a species with a large brain sustaining the necessary complex computations and other complex cognitive features—those that were no option for tiny bees [17].

## Data Availability

All the computer files employed in this study can be found at https://github.com/andszilagyi/ELBA_model. The data are provided in the electronic supplementary material [52].
